# A Novel Combined Soft Tissue and Bony Repair of Trochanteric Fractures in Revision Hip and Periprosthetic Fractures—Greater Trochanteric Abductor Tendon Augmentation (GTATA)

**DOI:** 10.3390/mps9010019

**Published:** 2026-01-28

**Authors:** Nina Handzewniak, Abid Mahmood, Canan Metin, Shahnawaz Khan, Tanvir Khan, Henry Atkinson

**Affiliations:** 1 Royal Free London NHS Foundation Trust, Pond Street, London NW3 2QG, UK; 2 Maidstone and Tunbridge Wells NHS Trust, Tonbridge Road, Royal Tunbridge Wells TN2 4QJ, UK; 3 Royal National Orthopaedic Hospital NHS Trust, Brockley Hill, Stanmore HA7 4LP, UK

**Keywords:** abductors insufficiency, abductors repair, greater trochanter fracture, greater trochanter fixation, novel surgical technique

## Abstract

Introduction: Management of trochanteric fractures in revision hip surgery has a high incidence of non-union and complications. Fixation devices are often bulky, prone to breakage, and necessitate reoperation. This study describes a novel soft tissue and bony abductor repair that reduces the forces on bony fragments without the need for prominent metalwork. Methods: This novel surgical technique involves fixation of the abductor mechanism with polyester and polyethylene sutures that are woven through the abductors and secured to the femoral shaft with a proprietary suture cerclage tape with cerclage wire supplementation in select cases. All patients undergoing fixation were retrospectively reviewed with a minimum follow-up period of 12 months. Outcomes relating to dislocation, reoperation, fracture union and the incidence of symptomatic Trendelenburg gait were recorded. Results: A total of 17 patients underwent this novel intervention. There were no dislocations or reoperations for prominent metalwork at the last follow-up. One patient had evidence of greater trochanter (GT) non-union, and three had GT displacement of over 3 mm. Eight (47.1%) patients were independently mobile and seven (41.2%) were mobile with only one walking aid. No patients required plate or bolt fixation. Conclusions: GT fractures and abductor deficiency are difficult to manage, with most reported methods utilising bulky metalwork to treat a soft tissue injury. We describe a novel combined soft tissue and bony fixation without the need for excessive metalwork. Our pilot study demonstrates satisfactory outcomes of this intervention that are technically reproducible and more appropriately addresses the deforming forces involved with a low complication profile.

## 1. Introduction

Insufficiency of the abductor mechanism of the hip is a life-altering complication associated with hip surgery and traumatic hip fractures. The insufficiency can result from bony or soft tissue deficiency, or often a combination of both [1]. The main hip abductors include gluteus medius (Gmed), gluteus minimus (Gmin) and tensor fascia lata [2]. Common pathologies involving those structures include abductor tendinopathy and complete or partial rupture of abductor tendons. The latter group often involves pathologies of the greater trochanter (GT), which is a site of insertion of two major abductors, Gmed and Gmin, and includes GT fractures in a native hip, periprosthetic hip fractures, and intertrochanteric neck of femur fractures (NOFs). Finally, some patients might suffer from abductor insufficiency due to iatrogenic causes, such as patients who require trochanteric osteotomy or complex/revision hip arthroplasty [3].

In the setting of hip arthroplasty, the management of abductor tendon injury depends on the underlying aetiology, as well as patient-related factors such as co-morbidities and mobility [4]. Patients with a mismanaged abductor injury can develop multiple complications, such as hip dislocation, persistent lateral hip pain, Trendelenburg gait and difficulties mobilising, which emphasises the need for an appropriate treatment choice [1,3,4]. Simple GT fractures in a native hip, as well as abductor tendinopathies, can often be managed in a conservative fashion, whereas more complex injuries will likely require surgical repair.

The surgical intervention of choice in GT fractures and abductor tendon injury is dependent on bone stock, the location of the injury and the surgeon’s training for such injuries [5].

Despite a number of validated techniques in managing them, such injuries’ outcomes remain variable, with a high complication profile [6].

Isolated suture repair of the abductor mechanism has a high failure rate due to the relatively low ability of the sutures to resist the forces involved in hip abduction [3]. Cerclage wires have been shown to provide greater stability and resistance to tension; however, they still carry a high risk of metalwork failure and/or breakage, as well as the risk of persistent pain [6]. Finally, more complex repair systems, such as cable plate systems or locking plates, provide better fixation but are prone to soft tissue irritation with an increased incidence of infection, MW failure and further revision surgeries [7]. Furthermore, the above techniques often only focus on the fixation of one component of the abductor mechanism (i.e., bony fixation or soft tissue repair), which fails to address it as a one structure. The challenge remains to be controlling the thin cancellous GT fragments which are subject to large abductor tendon forces. Late displacement or cutting out of metalwork is not uncommon. Moreover, fixation can potentially amplify the fragmentation of bone.

The aim of this study is to describe a novel combined soft tissue and bony repair technique, in the setting of a GT fracture or abductor deficiency, and assess its efficacy.

## 2. Methods

### 2.1. Surgical Technique

**Figure 1:**
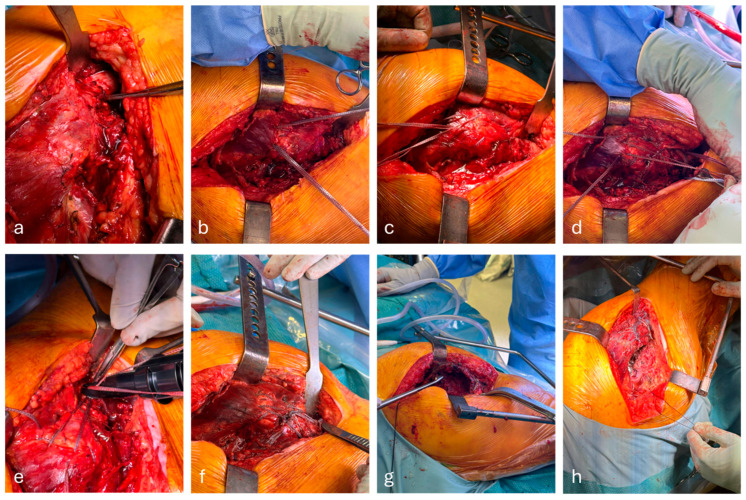
Step by step illustration of abductor repair, using Arthrex FiberTape. Abductor deficiency visualised in a patient with an intertrochanteric neck of femur fracture (**a**). FiberTape weaved through the proximal abductors (**b**). Double whipstitch weaved along the edges of the abductors, gaining control of the abductor tendon (**c**). Cerclage wire tensed in a figure of eight, passed through an interosseus, tunned in the greater trochanter (**d**). FiberTape cerclage tensioned around femoral shaft with proximal FiberTape passed through the tensioner loop. It is essential to tie the proximal FiberTape while the FiberTape cerclage is held under tension (**e**). Complete reconstruction of abductors visualised (**f**). Femoral canal prepped for cemented hemiarthroplasty (**g**). Final construct demonstrating abductor repair with a cemented hemiarthroplasty stem in situ (**h**).

This is separated into both a bony and soft tissue repair (Figure 1).

#### 2.1.1. Soft Tissue:

All patients underwent abductor repair, using a novel combined soft tissue and bony technique. Two separate sutures made of ultra-high molecular weight polyethylene (UHMWPE) and polyester weave (Arthrex FiberTape) were pulvertaft weaved into the common abductor tendon (Figure 1b,c), where comminuted bony fragments are present. These are incorporated into the weave through soft tissue attachments. This should leave 4 limbs—2 posterior and 2 anterior—giving complete control of the abductor complex.

#### 2.1.2. Bony Fixation

**Figure 2:**
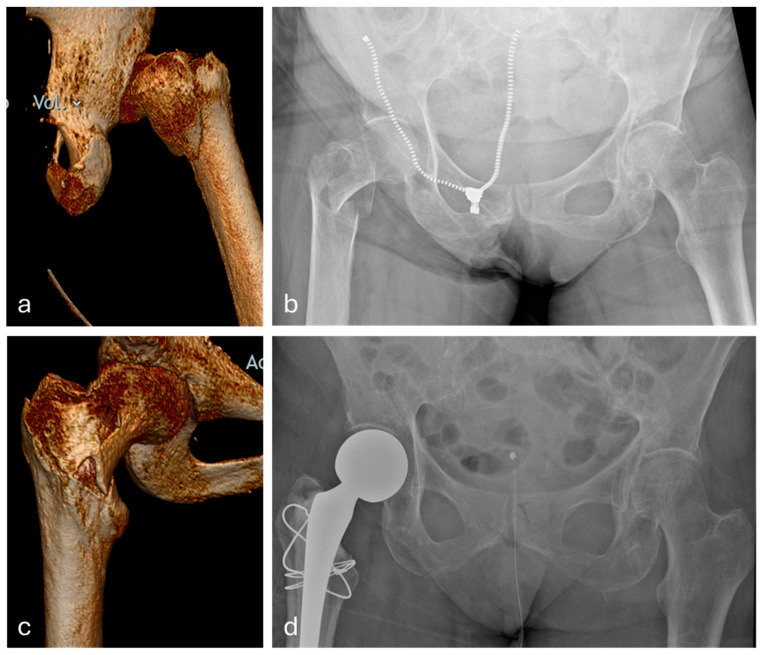
Pre and post op radiological images from a 77-year-old female patient whose abductor reconstruction was visualised in Figure 1. Three-dimensional CT visualisation of the right intertrochanteric neck of femur fracture (**a**,**c**). Pelvic radiograph demonstrating the same right-sided neck of femur fracture on admission (**b**). Pelvic radiograph post hemiarthroplasty and abductor reconstruction (**d**).

Bony fixation is predicated on the size of GT fragment. If fragments are larger than 2 cm^2^ and do not pass the vastus ridge, then a 2.5 mm Pilot hole is utilised across the fragment and a figure of 8 of 1.6 mm cerclage wire is passed through the bony fragment and wrapped under the LT, forming a figure of 8, and tensioned with the wire knot placed on the anterolateral aspect of the femur (Figure 1d). This process can be doubled for larger fragments. The crossing of the wires should rest over the fracture site (Figure 2). It is crucial to avoid over-tensioning, due to the risk of cut out.

#### 2.1.3. Soft Tissue Fixation

A separate strand of UHMWPE and polyester tape (Arthrex FiberTape Cerclage) was looped four times around the femoral shaft. This suture has a tensionable loop. Two free ends form the FiberTape within the proximal tendon and are then passed through the tensionable loop. The loop is tensioned, while the FiberTape ends are placed under traction to reduce the abductor tendon. While the cerclage tape is tensioned and held on the tensioner, the two proximal FibreTapes are tied (Figure 1e). The cerclage loop is then secured with a simple surgeons’ knot. The knot should sit just inferior to the previous cerclage wire to prevent proximal sliding of this fixation. The remaining free ends are then passed again in a figure of 8 around the proximal femur and passed through a second cerclage tape and tensioned, resulting in a secure fixation, resulting in both the abductor tendon and the GT gaining fixation points on the distal femur.

This technique obviates the need for anchors and combines a bony and soft tissue repair without the need for bulky plates and cables and is then prone to loosening and failure. For larger fractures, second suture cerclage is placed just inferior to the LT to tension, and any remaining fracture gap is closed. For patients with only abductor avulsion, the cerclage wire is not required. In patients with a Gt fragment at or distal to the Vastus ridge, a cerclage wire is also employed in a figure of 8 fashion along with FibtreTape tendon augmentation.

### 2.2. Patient Cohort

This single-centre, two-surgeon study included a retrospective analysis of a group of 17 patients who underwent surgical fixation of their abductors, using the technique described above. The patients included were all over 18 years old (62–91) and required abductor fixation due to one of the following pathologies: abductor failure post total hip replacement (THR); revision for a periprosthetic fracture–THR; revision for a periprosthetic fracture–DHS; abductor failure with dislocation post hemiarthroplasty; proximal femoral replacement dislocation; intra-operative GT fracture–hemiarthroplasty; intra-operative GT fracture–THR and complex primary THR for trauma. This study is a proof-of-concept paper, hence the low number of included patients. It primarily reports a technique, rather than outcomes. Validated outcomes are needed to reaffirm the presented findings.

All patients were followed up locally for at least 12 months, undergoing regular clinical and radiological assessment. The mean follow-up period was 15 months (12–28 months). The inclusion criteria were broad but patients with infection, previous use of synthetic graft or marked osteolysis and those with a high chance of bleeding were all excluded. The patients included had both acute and chronic injuries.

### 2.3. Analysis

Patient data were obtained from the Electronic Patient Record and Picture Archiving and Communication System. Each patient was assessed for occurrence of the following complications: wound complication, hip dislocation, revision for metalwork irritation, GT displacement from immediately post-operation, non-union of the GT, metalwork failure, presence of Trendelenburg gait and use of walking aids. The presence of Trendelenburg gait was performed in clinic as part of a routine clinical examination.

### 2.4. Radiological Assessment

All imaging was reviewed by two senior Orthopaedic surgeons pre- and post-injury. Calibrated radiographs allowed for the measurement of GT displacement in mm. The mean of the two figures were taken. Displacement was judged as vertical movement with 3+ mm from the GT’s original location, recorded as displaced. These recordings were taken within 24 h of admission and 3 months after fixation [8]. Union was judged as cortical bridging bone between the displaced GT fragment and the lateral shaft of the femur.

### 2.5. Ethics

All patients consented to medical photography. This was a two-surgeon retrospective study, thus nominally falling within the remit of service evaluation. Such studies are not required by the UK Integrated Research Application System (IRAS) to be submitted for ethical approval. All patient data were blinded from the end users. No AI tools were used during the study design, data analysis or manuscript preparation. None of the authors have any financial holdings in the companies related to this study, with no conflicts of interest. The study was not funded. The study was recorded with the departmental research lead, who is also a co-author in the study.

## 3. Results

### 3.1. Demographics

The analysed cohort included 12 females and 5 males, with ages ranging from 62 years to 91 years at the time of surgery. A total of 16 out of the 17 patients were emergency trauma cases. The indications for the fixation of the abductor mechanism were as follows: abductor failure post-total hip replacement (one patient), revision for a periprosthetic total hip replacement fracture (four patients), revision for a periprosthetic DHS-related fracture (two patients), abductor failure with dislocation post-hemiarthroplasty (two patients), proximal femoral replacement dislocation (one patient), intra-operative GT fracture–hemiarthroplasty (one patient), intra-operative GT fracture–total hip replacement (two patients) and complex primary total hip replacement for trauma (four patients).

### 3.2. Rate of Complications

All patients in the analysed cohort were followed up for a minimum of 12 months (ranging from 12 to 18 months). Within the follow up period, 2/17 (11.8%) of patients developed conservatively managed wound complications, 0/17 (0%) developed a dislocation, 0/17 (0%) underwent metalwork removal, 3/14 (21.4%) had evidence of GT displacement of at least 3 mm when compared to post-operative radiographs, 1/14 (7.1%) had evidence of GT non-union, 1/15 (6.6%) suffered from metalwork failure, 4/17 (23.5%) developed Trendelenburg gait and 11/17 (64.7%) required at least one walking aid (Table 1).

## 4. Discussion

This study demonstrates a novel technique for combined soft tissue and bony abductor repair and assesses its efficacy in a patient cohort with various abductor pathologies. The technique involves the fixation of abductor tendons with a polyethylene–polyester weave, which is then tensioned to the femoral shaft, as well as additional fixation of the bony elements with a figure of eight tension wire. As a result, the created construct is strong enough to provide structural support to the damaged abductor mechanism, and at the same time remains compact enough to avoid tissue irritation and persistent pain.

Within the observed cohort follow-up period, two patients developed minor wound complications that did not require a return to theatre and three had evidence of GT displacement of at least 3 mm on the radiograph when compared to immediate post-operative imaging, but only one of these developed a GT non-union. Only one in five patients demonstrated an ongoing abductor gait. Nine of the patients required at least one walking aid 12 months post-surgery, but eight of these were using aids pre-operation and had returned to their pre-operative status. Only one of the patients developed metalwork failure (breakage of metalwork), and one had evidence of GT non-union. None of the patients had to undergo metalwork removal or experienced dislocation.

The above technique is a new alternative to abductor fixation, addressing common complications associated with current fixation techniques. These can be broadly divided into use of interosseus sutures alone, fixation with cerclage wires, cable systems, or use of locking plates. Each of those groups presents different challenges and can be associated with various complications.

The use of interosseous sutures alone is a widely available technique and provides a reasonable option for the fixation of soft tissues only [2]. It involves the fixation of completely or partially ruptured tendons to the greater trochanter, using either interosseous tunnels or suture anchors. The advantages of this technique are its low cost, low complexity and short operating time [9,10]. However, the evidence behind its efficacy is very varied, with some studies reporting up to a 25% failure rate. There is also no clear consensus regarding the use of tunnels versus anchors, or single versus double rows of sutures [2,11], which makes the technique difficult to standardise and thus might lead to inconsistent outcomes. Additionally, anchors are challenging to deploy in the presence of implants and the periprosthetic setting.

Cerclage and tension band wires are some of the most popular tools to fix abductor deficiency with bony involvement. Similarly to the sutures, they are widely available at a low cost and are simple to use. They are considered to provide stronger fixation than using sutures alone, and many studies suggest better outcomes in terms of persistent pain when compared to other metal fixation devices [12]. Furthermore, the simple design allows for versatility in use and combining with other fixation devices. On the other hand, the use of wires alone has a high rate of non-union (up to 30%) and wire breakage and migration [10].

Cables are another alternative for GT fixation, with evidence for lower rates of non-union than sutures alone [13]. However, multiple studies report a risk of fracture non-union of up to 30% when using cables alone and thus, they are frequently used as part of a cable-claw plate fixation system [10]. Cadaveric studies suggest that cable systems can resist significantly higher lateral and vertical forces than cerclage wires alone [14]. However, in vivo studies are more contradictory to each other when analysing cable use. A study directly comparing the use of figure of eight wires and cable-claw plate systems in patients found no significant difference in union and osteolysis rates but noted that the cable systems were associated with a four times higher incidence of persistent pain [12]. Multiple other studies report that cable–plate systems are associated with non-union rates between 12% and 24%, and the need for revision required in up to 10% of patients [6,15]. Furthermore, even though the cable–plate systems are less bulky than standard locking plates, many patients still develop persistent pain and plate-related complications [16,17].

Finally, the use of locking plates is considered to be the most invasive option but provides the strongest fixation out of all available methods. The rates of non-union associated with the locking plates are reported to be less than 10% [18] and the rates of metalwork failure are at the level of 15%, compared to the nearly 30% reported for cables [15,16]. Locking plates can also be used in revision procedures for GT non-union, achieving over 85% union rates radiologically [18]. However, the main issue reported with locking plates is the high rates of persistent lateral hip pain, which are reported to affect up to 20% of patients and are associated with the need for plate removal.

The aim of the technique presented in this study is to avoid the complications associated with use of bulky metalwork, such as pain and high revision rates, while maintaining high resistance to tensile abductor forces, which is not achieved with use of sutures alone (Table 2). The polyethylene–polyester weave whip stitch suture provides a reliable method for soft tissue repair, and fixation with looped and tensioned cerclage tapes tackles the issue of secure tendon attachment to the bone. Use of cerclage wires, tied in a figure of eight fashion, additionally strengthens the construct and provides bone-to-bone fixation. As a result, the system allows for a strong fixation, while maintaining low bulk to avoid soft tissue irritation and persistent pain. The efficiency of this technique is backed up by the results of this study, which demonstrated low levels of post-operative complications such as metalwork failure or the need for metalwork removal, at the same time demonstrating good abductor function in over 75% of the patients.

In conclusion, the above technique offers an alternative to conventional abductor repair techniques, providing a strong fixation while aiming to reduce some of the commonly encountered complications.

### Study Limitations

The limited sample size of 17 makes firm conclusions difficult to draw. Moreover, indications were varied, with the technique being applicable to a broad number of pathologies with a heterogenous patient cohort. This supports the wide-ranging efficacy of the technique but makes the external application of the study results more challenging. This is a proof-of-concept study; thus, it forms the basis for further, wider-reaching research into this novel technique. Given the small sample size, further sub-group analysis is of limited value. In relation to outcome measures, the primary end-point was bony union or tendon healing; going forward, comparisons between groups with validated outcome cores are required.

## 5. Conclusions

Abductor tendon or GT fractures around hip prosthesis remain a challenging pathology to manage. The magnitude of the abductor-deforming force results in bony fixation often failing. Gaining control of the abductor tendon and neutralising the deforming forces is not possible with metalwork fixation only. Soft tissue repair is weakened by poor methods to hold the sutures with limited real estate for bony anchors. We present a novel combined greater trochanter, abductor tendon augmentation repair (GTATA) with both soft tissue and bony repair. This technique overcomes the shortcomings of many of the hitherto described interventions addressing both aspects of the injury, obviating the need for bulky metalwork. In this single-centre cohort, we demonstrated high rates of union and improved long-term mobilisation.

## Figures and Tables

**Table 1:** Indication for abductor repair and complications developed at last follow up. Numbers in the left-hand column indicate each individual patient. Age at the time of the procedure given in years. M—male; F—female; N—no; Y—yes; .—answer not applicable; THR—total hip replacement; PPF—periprosthetic fracture; DHS—dynamic hip screw and PFR—proximal femoral replacement.

Patient Number	Age	Gender	Indication	Wound Complication	Dislocation	Aids at 12 Months	Re-Operation for Metal Irritation	GT Displacement from Post Op	Union of GT	Metalwork Failure Fracture	Trendelenburg
**1**	64	M	**Abductor failure post THR**	N	N	Nil	N	.	.	N	N
**2**	71	M	**PPF-THR**	N	N	1 stick	N	N	Y	N	Y
**3**	89	F	**PPF-THR**	N	N	1 stick	N	Y	N	Y	N
**4**	65	F	**PPF-THR**	N	N	1 stick	N	N	Y	N	N
**5**	78	F	**PPF-THR**	Y	N	Nil	N	N	Y	N	N
**6**	81	F	**PPF-DHS**	N	N	2 Stick	N	N	Y	N	Y
**7**	73	F	**PPF-DHS**	Y	N	Nil	N	N	Y	N	N
**8**	88	F	**Abductor failure with dislocation-hemiarthroplasty**	N	N	Frame	N	.	.	.	Y
**9**	89	F	**Abductor failure with dislocation-hemiarthroplasty**	N	N	1 stick	N	.	.	.	N
**10**	73	F	**Dislocation PFR**	N	N	Nil	N	Y	Y	N	N
**11**	91	F	**Intra-op GT fracture Hemiarthroplasty**	N	N	1 stick	N	N	Y	N	N
**12**	70	F	**Intra-op GT fracture THR**	N	N	Nil	N	N	Y	N	N
**13**	78	M	**Intra-op GT fracture THR**	N	N	Nil	N	N	Y	N	N
**14**	83	F	**Complex Primary THR for Trauma**	N	N	1 Stick	N	N	Y	N	N
**15**	72	F	**Complex Primary THR for Trauma**	N	N	Nil	N	Y	Y	N	N
**16**	74	M	**Complex Primary THR for Trauma**	N	N	Nil	N	N	Y	N	N
**17**	85	M	**Complex Primary THR for Trauma**	N	N	1 stick	N	N	Y	N	Y

**Table 2:** The advantages of the described technique over established interventions. GTATA—Greater trochanteric abductor tendon augmentation.

Intervention	Metalwork Failure or Breakage	Need for Removal	Soft Tissue Repair	Risk of Soft Tissue Irritation	Failure of Fixation	Complexity
Plate and Cables	Low	High	No	High	Low	High
Cerclage Wires	High	High	No	High	High	Low
Sutures	/	Low	Yes	Low	High	High
Hook Plates	Low	High	No	High	High	Low
GTATA	Low	Low	Yes	Low	Low	Low
Synthetic Graft Jacket	Low	High	Yes	High	High	High

## Data Availability

The original contributions presented in this study are included in the article. Further inquiries can be directed to the corresponding authors.
